# Screening for oral potentially malignant disorders among areca (betel) nut chewers in Guam and Saipan

**DOI:** 10.1186/1472-6831-14-151

**Published:** 2014-12-11

**Authors:** Yvette C Paulino, Eric L Hurwitz, Saman Warnakulasuriya, Robert R Gatewood, Kenneth D Pierson, Lynnette F Tenorio, Rachel Novotny, Neal A Palafox, Lynne R Wilkens, Grazyna Badowski

**Affiliations:** Cancer Research Center of Guam, University of Guam, UOG Station, Mangilao, GU USA; University of Hawai’i, Cancer Center, 701 Ilalo Street, Honolulu, HI USA; King’s College London Dental Institute and WHO Collaborating Centre for Oral Cancer, London, UK; Reflection Center Suite 303, 222 Chalan Santo Papa, Hagatna, GU USA; Saipan Seventh-Day Adventist Clinic, Quartermaster Road, Chalan Lau Lau, Saipan, MP USA; P.O. Box 500027, Saipan, MP USA

**Keywords:** Areca, Betel, Guam, HPV, Mariana Islands, Micronesia, Oral potentially malignant disorders, Oral precancer, Saipan

## Abstract

**Background:**

The Mariana Islands, including Guam and Saipan, are home to many ethnic subpopulations of Micronesia. Oral cancer incidence rates vary among subpopulations, and areca (betel) nut chewing, a habit with carcinogenic risks, is common. Our objectives were to conduct a screening program to detect oral potentially malignant disorders (OPMD) in betel nut chewers, measure their betel nut chewing practices, and assess the prevalence of the oral human papillomavirus (HPV) infection in a subset of betel nut chewers in these islands.

**Methods:**

A cross-section of 300 betel nut chewers ≥18 years old [in Guam (n = 137) and in Saipan (n = 163)] were recruited between January 2011-June 2012. We collected demographic, socioeconomic, and oral behavioural characteristics. Latent class analysis was used to identify chewing patterns from selected chewing behaviours. Following calibration of OPMD against an expert, a registered oral hygienist conducted oral examinations by house to house visits and referred positive cases to the study dentist for a second oral examination. Buccal smears were collected from a subset (n = 123) for HPV testing.

**Results:**

Two classes of betel nut chewers were identified on 7 betel nut behaviours, smoking, and alcohol use; a key difference between the two Classes was the addition of ingredients to the betel quid among those in Class 2. When compared on other characteristics, Class 1 chewers were older, had been chewing for more years, and chewed fewer nuts per day although chewing episodes lasted longer than Class 2 chewers. More Class 1 chewers visited the dentist regularly than Class 2 chewers. Of the 300 participants, 46 (15.3%; 3.8% for Class 1 and 19.4% for Class 2) had OPMD and one (0.3%) was confirmed to have squamous cell carcinoma. The prevalence of oral HPV was 5.7% (7/123), although none were high-risk types.

**Conclusions:**

We found two patterns of betel nut chewing behaviour; Class 2 had a higher frequency of OPMD. Additional epidemiologic research is needed to examine the relationship between pattern of chewing behaviours and oral cancer incidence. Based on risk stratification, oral screening in Guam and Saipan can be targeted to Class 2 chewers.

## Background

Areca nut is the fruit of the *Areca catechu* tree that contains alkaloids (particularly arecoline) and tannins, which are the nut’s most active ingredients. In most of the world, the nut is habitually chewed with other ingredients (e.g. leaf from *Piper betle*, slaked lime, tobacco and spices). When the nut is chewed with other ingredients, generally betel leaf, lime, with or without tobacco, it is called a betel quid. Approximately 10-20% of the world’s population chews areca (betel) nut [1].

The alkaloids and nitrosamines in areca nut are considered carcinogens, and could produce precancerous lesions or conditions – now referred to as potentially malignant disorders – that are likely to develop into oral cancer [2]. In 1987, the International Agency for Research on Cancer (IARC) concluded that chewing betel quid containing tobacco was carcinogenic to humans [3]. In 2004, the IARC revised their evaluation that betel quid with and without tobacco and areca nut by itself were carcinogenic to humans. The Working Group specifically noted the following: 1) there was sufficient evidence in humans and experimental animals for the carcinogenicity of betel quid with and without tobacco; 2) there was sufficient evidence in experimental animals for the carcinogenicity of areca nut, and 3) there was evidence suggesting lack of carcinogenicity in experimental animals for betel leaf and slaked lime [2].

Fortunately, the oral cavity is one of few sites of the human body in which early signs of cancer can be detected by visual examination [4]. The changes that precede the development of oral cancer are known as precancers. In 1978, the World Health Organization (WHO) classified precancers into lesions (leukoplakia, eythroplakia, palatal lesions in reverse smokers) and conditions (lichen planus, submucous fibrosis, discoid lupus erythematosus, syphilis, sideropenic dysphagia, actinic keratosis). Today, these are grouped together as potentially malignant disorders [5].

Because areca nut is often chewed with betel leaf, it is known in many places as betel nut. This is especially true in Micronesia, and thus the term “betel nut” will be used to refer to any form of areca nut use for the remainder of this paper. The preparation and consumption of betel nut in Micronesia differs among ethnic communities in the type of nut used, ingredients added, and whether or not ingestion takes place [6, 7]. Two statistically distinct groups of betel nut chewers have been identified in Guam [7]. One group includes predominantly native Chamorros of Guam. This group chews the red, mature nut by itself and ingests the nut. Some chewers in this group would occasionally add betel leaf. The second group includes predominantly other Micronesians who have migrated to Guam. This group prefers to chew a custom-made betel quid, which includes the unripe nut, betel leaf, slaked lime, and tobacco (often from a cigarette stick). Unlike the first group, the chewers in the latter group often spit out the betel quid and juices.

The risk of oral cancer is higher in Micronesia relative to the United States. The period prevalence (1985–1998) of oral cancer, age-adjusted to the 1988 U.S. population, in Micronesian islands was (in descending order per 100,000 population): 31.8 in Yap, 16.2 in the Marshall Islands, 16.1 in Palau, 13.1 in Kosrae, 7.7 in Pohnpei, and 4.2 in Chuuk [8]. In Guam, the incidence (1997–2003) of mouth cancer, age-adjusted to the 2000 U.S. population, was (in descending order per 100,000 population per year): 17.9 for other Micronesians, 8.1 for Chamorros, 5.5 for Whites, 3.6 for Asians, and 2.3 for Filipinos [9].

Haddock and colleagues (1981) examined betel nut chewing and other risk factors associated with oral cancer in a cross-sectional sample of Guam residents [10]. The authors concluded that both smoking and betel nut use were significantly associated with oral disease, and were equal in their degree of association. The study provided useful data on descriptive patterns of betel nut usage and precancerous lesions; however, the scope was limited in that the intensity of the risk factor behaviours (alcohol use, betel nut use and smoking) were not quantified.

The practice of betel nut chewing extends to adolescents and children. Results from a 1995 survey conducted in Palau indicated that 55% of children 5–14 years of age chewed betel nut [11]. As a result of that study, a bill was passed in the Republic of Palau Senate in 2011 prohibiting the use and sale of betel nut to minors [12]. In the Commonwealth of the Northern Mariana Islands (CNMI), in Saipan, a high prevalence (63.4%) of regular betel nut use was documented in a cross-sectional survey of high school students [13] - the highest prevalence ever recorded for betel nut use in a school population survey. The study also revealed that 13% of the children who participated had oral leukoplakia and 8.8% had oral submucous fibrosis, both of which are potentially malignant diseases or disorders.

The purpose of this study was to pilot test the methods for studying oral potentially malignant disorders (OPMD) in betel nut chewers in Guam and Saipan in Micronesia. Specifically, we aimed to conduct a screening of OPMD in betel nut chewers, measure the betel nut chewing practices of those chewers, and assess the prevalence of oral human papillomavirus (HPV) infection in a subset of betel nut chewers in the study.

## Methods

Approvals from the Institutional Review Boards at the University of Hawaii-Mānoa (CHS #18174) and the University of Guam (CHRS #10-73) were obtained. All the participants were informed of and consented to the study.

### Site selection

A cluster sampling approach was used to identify groups of betel nut chewers in two of the islands in the Marianas; Guam and Saipan. Guam is a U.S. territory in the Western Pacific. Saipan is part of the CNMI, which established a commonwealth in political union with the U.S. Given that betel nut chewers in the Marianas region tend to be of Chamorro or other Micronesian Island ancestry [6], we focused recruitment on the villages (clusters) in each island with the highest population of natives according to the 2000 U.S. Census. Five villages (Inarajan, Merizo, Sinajana, Talofofo, Umatac) in Guam, and six villages (Kagman, Koblerville, Garapan, Oleai, San Antonio, Tanapag) in Saipan were selected.

### Recruitment

Recruitment to study was from January 2011 to June 2012, using several strategies where the sampling was purposive conditioned on chewing habit. In Saipan, households and participants were selected within village clusters, using a method employed in previous studies [14, 15]. An object with a pointed end was spun at the village cluster center to determine the direction to proceed. Every house in that direction was approached for recruitment. To be eligible, an individual had to be: 1) at least 18 years of age, 2) a betel nut chewer, and 3) willing to consent. Up to three eligible participants per household were invited to participate. All eligible candidates within a household were invited with an upper limit of three participants/household; in cases where there were more than three a die was used to randomly select the three participants.

The recruitment on Guam was initially similar to Saipan; however, possibly due to the lower betel nut chewing prevalence in Guam than in Saipan (12% versus as high as 90% [16]), recruitment was slower in Guam. Furthermore, gates and dogs at many homes made house-to-house recruitment in the first village in Guam a particular challenge. Only 16 participants were recruited through this method. Unlike the island of Saipan where there is only one Mayor, each village has a Mayor on the island of Guam who maintains a registry of households. Although the registers did not include betel nut chewing habits, the Village Mayors used their familiarity with the residents to help the research team identify households from the registry list where they believed betel nut was chewed. This method resulted in 67 additional study participants. After the recruitment through village mayors was exhausted, a request was submitted to the Institutional Review Boards to modify recruitment to a convenience sample. The remaining 54 participants (39% of the total Guam sample) were recruited through advertisements (n = 22) and community gatherings (n = 32) from the rest of the island.

### Survey

Two teams conducted the survey, one in each island. The teams consisted of a lead dentist, a registered dental hygienist, and two interviewers. The dental professionals performed the oral screening for OPMD, and they collected oral brush samples to screen for HPV infection. The interviewers administered the survey questionnaires.

### Questionnaires

The questionnaires used in this study include information on demographics, socioeconomic status, and betel nut chewing behaviours. A validated betel nut Questionnaire was available to the study group [17] and this was modified for this study. All the betel nut questions with substantial to almost perfect agreement, where κ = 0.61 to 1.0 [18], in the validation study were retained. The questions on slaked lime and betel leaf usage frequency, which scored low in the validation study, were simplified to dichotomous responses.

### Oral screening

Prior to conducting any surveys, the research teams participated in a two-day training session led by a WHO expert (SW) familiar in the detection of OPMD in betel nut chewers. On the first day, the teams reviewed the protocols for performing a 3-minute oral screening [19]. Thereafter, the dentists and hygienists were calibrated against the expert on the detection of OPMD with numerous clinical illustrations. A percent agreement was calculated for each dentist and hygienist. The median percent agreement was 95% for risk status assessment and 80% for diagnosis.

During data collection, the oral screening was conducted in two tiers. The initial oral screening was conducted in the field (by house-to-house visiting) by the dental hygienist. If there was a suspect lesion or condition, as defined by the protocol, the dental hygienist referred the participant to the lead dentist. The second oral examination was conducted by the lead dentist at the clinic. The lead dentist collected biopsies, as needed, and sent the samples to a pathology laboratory for verification.

### Sample collection for HPV analysis

A subset of the participants was screened for the HPV. The 123 participants screened for HPV included every third participant, as well as those referred for a second oral screening. Those secreened for HPV were similar in demographics and chewing characteristics to those not screened for HPV. An oral sample was collected using similar protocols to those described by Hernandez and colleagues [20]. The registered hygienist used a Cytobrush plus Cell Collector ([product #1101] Medscand Medical AB, Sweden) to brush the lining of the mouth. All areas of the mouth were swabbed with the Cytobrush in a systematic fashion, including any screen-detected lesion. The brush was inserted into a transport tube of 1.0 mL buffered medium (Digene Corp., Gaithersburg, MD) and stored at -20°C until it was ready to be shipped. All of the samples were sent to the University of Hawaii Cancer Center, where they were tested for the presence or absence of HPV by consensus PGMY09/11 [21]. The positive samples were further tested for HPV Deoxyribonucleic acid (DNA) genotype by using linear array Roche line-blot test for 37 HPV types [22].

### Analyses

Previously, Paulino and colleagues [7] had statistically identified two chewing patterns among a small number (n = 49) of betel nut chewers in Guam. The analysis was replicated in this study with a larger sample of betel nut chewers (n = 300) to verify if any distinct patterns of betel nut chewing exist in the Mariana Islands. Mplus® software (Version 3, Los Angeles, California) was used to perform latent class analysis, a statistical modeling method to evaluate the relationship in categorical data where latent (unobserved) variables are identified from observed variables [23], in this case, patterns of betel nut chewing. The latent class model uses independent variables (continuous and categorical) to assign membership to a set number (*k*) of groups by maximum likelihood while adjusting for covariates [24]. The variables used to identify patterns of betel nut chewing were: betel nut variety (coded red, white, or both); betel nut maturity (coded unripe, ripe, or both); addition of betel leaf, calcium hydroxide (slaked lime), tobacco, alcohol (each coded yes or no); ingestion of betel quid (coded yes or no); and smoking and alcohol use (not as part of quid, each coded yes or no). Smoking was defined as the current use of cigarettes at least once per day. Alcohol use was defined as any consumption within 30 days prior to the interview. The analysis was adjusted for age and sex. Each individual in the study was assigned to one of the *k* classes based on the maximum of the conditional probabilities for each class estimated from the latent class model using the selected variables.

The IBM SPSS® software (Version 20, Armonk, New York) was used for statistical analyses. The F test was used to compare means and the Chi-square test was used to compare frequencies of characteristics between the two classes of betel nut chewers.

## Results

We recruited three hundred betel nut chewers - from Guam (n = 137) and Saipan (n = 163), for this study. Among the people approached through our house-to-house recruitment almost 100% complied, only one person refused to volunteer int eh study. Difficulties in identifying chewers in Guam are presented in the methods section, this mainly being inaccessibility to their front doors. There were a few refusals among community gatherings, but it was difficult to quantify the number of refusals in that setting.

### Classes of betel nut chewers

We found several varities of betel nut use. The two-class model was found to fit better than the three-class model in the latent class analysis, resulting in the identification of two classes of betel nut chewers. The classes were compared on the variables used to perform the latent class analysis (Table 1).

**Table 1:** **Comparison of variables used in the latent class analysis of betel nut chewers**, **by chewing class**

	Class 1 n = 78	Class 2 n = 222	***P*** value*
	n (%)	n (%)	
Betel nut maturity**			<0.001*
% that chew young betel nut	3 (3.8)	183 (82.4)	
% that chew mature betel nut	73 (93.6)	18 (8.1)	
% that chew both equally	0	21 (9.5)	
Betel nut variety			<0.001*
% that chew red variety	71 (91.0)	118(53.2)	
% that chew white variety	3 (3.8)	79 (35.6)	
% that chew both equally	4 (5.1)	25 (11.3)	
% that add betel leaf	44 (56.4)	172 (77.5)	<0.001*
% that add calcium hydroxide (lime)	3 (3.8)	222 (100)	<0.001*
% that add tobacco	11 (14.3)	194 (87.4)	<0.001*
% that spike ingredients with alcohol	6 (7.7)	19 (8.6)	0.81
% that swallow betel quid	74 (96.1)	71 (32.0)	<0.001*
% that smoke	46 (59.0)	93 (42.1)	0.01*
% that consume alcohol	45 (57.7)	132 (59.5)	0.79

n = number.

*Reflects statistical difference between Classes, where *P*<0.05.

**Includes two participants unsure of betel nut maturity consumed.

The majority of Class 1 chewers preferred the mature (93.6%) betel nut in red only (91.0%), white only (3.8%), or both (5.1%) varieties. Very few Class 1 chewers preferred the young betel nut (3.8%), and none chewed both young and mature betel nuts equally. The majority of Class 2 chewers preferred the young betel nut (82.4%) in red only (53.2%), white only (35.6%), or both (11.3%) varieties. Few Class 2 chewers preferrd to chew both the young and mature betel nuts (9.5%), and even fewer preferred the mature betel nuts only (8.1%). Compared to Class 2, significantly fewer Class 1 chewers used betel leaf, calcium hydroxide (slaked lime), and tobacco with their betel nut. However, compared to Class 2, significantly more Class 1 chewers swallowed the by-products of the betel nut during chewing and smoked cigarettes.

The two classes of betel nut chewers were also compared on other characteristics (Table 2). The mean (95% CI) age was 37.7 (36.2 – 39.3) years, although Class 1 chewers were older than Class 2 chewers. The majority (52.3%) of the participants were males. Only a few (7.0%) had post-secondary education and about a third (32.7%) were married.

**Table 2:** **Comparison of other characteristics of betel nut chewers**, **overall and by chewing class**

	Overall	Class 1	Class 2	***P*** value**
	n = 300	n = 78	n = 222	
	X [ 95% CI] * or n (%)	X [ 95% CI] or n (%)	X [95% CI] or n (%)	
**Demographic/** **Socioeconomic characteristics**				
Age, years	37.7 [36.2-39.3]	45.7 [42.8-48.7]	34.9 [33.3-36.6]	<0.001**
Gender, % male	157 (52.3)	38 (48.7)	119 (53.6)	0.46
Education, % with post-secondary	21 (7.0)	3 (3.8)	18 (8.1)	0.20
Marital status, % married	98 (32.7)	29 (37.2)	69 (31.1)	0.32
**Behavioural characteristics**				
Length of betel nut use, years	19.8 [18.3-21.3]	25.5 [22.1-28.9]	17.8 [16.2-19.3]	<0.001**
Number of nuts chewed per day	12.8 [11.3-14.2]	7.3 [5.32-9.18]	14.6 [12.9-16.4]	<0.001**
Length of time betel nut chewed, minutes	18.7 [10.5-26.9]	37.8 [7.07-68.6]	12.0 [9.51-14.4]	0.01**
% that visit the dentist	89 (29.7)	35 (44.9)	54 (24.3)	<0.001**
**Oral Potentially Malignant Disorders**				
% screened positive***	46 (15.3)	3 (3.8)	43 (19.6)	<0.001**

n = number.

*X [95% CI] = Mean [95% Confidence Interval].

**Reflects statistical difference between Classes, where *P*<0.05.

***Oral Potentially Malignant Disorders detected during the initial oral screening.

Class 1 chewers reported chewing betel nut for more years than Class 2 chewers (25.5 years versus 17.8 years; p ≤ 0.01). Class 1 chewers also chewed fewer nuts per day (7.25 nuts versus 14.6 nuts; p ≤ 0.01), but their chewing episodes lasted longer than those of Class 2 chewers (37.8 minutes versus 12.0 minutes; p = 0.01).

More Class 1 chewers visited the dentist regularly compared to Class 2 chewers (44.9% versus 24.3%; p ≤ 0.01).

### Oral screening

All 300 participants underwent an initial oral screening during house to house visits. Forty-six (15.3%) were found to have suspect lesions during the initial screening. Some participants displayed multiple oral lesions and conditions. The majority of the subjects found during the initial screening had mixed red and white lesions (n = 26), followed by a white lesion (n = 16), submucous fibrosis (n = 5), ulcerated lesion (n = 3), and an exophytic lesion (n = 1). Of the 46 referred for a second oral exam, 27 (58.7%) attended the second oral examination; 22 of the 27 (81.5%) were confirmed to have OPMD. Assuming that 81.5% of all lesions found at initital screening would have been verified, the dentist-verified prevalence of OPMD is 12.4% (81.5%*46/300). Seven participants screened by the dentist underwent an oral biopsy; three were of mixed red and white lesions, two of white lesions, and one each of ulcerated lesion and an exophytic lesion. The exophytic lesion was confirmed to be squamous cell carcinoma. More Class 2 chewers had OPMD than Class 1 chewers (19.4% versus 3.8%; p ≤ 0.01) (Table 2). We have no data to explain the reasons for non-compliance by 19 subjects who did not attend the specialist.

### HPV

A total of 123 participants were tested for HPV, of which 7 (5.7%) were positive for HPV-DNA. One was found to have a low-risk type (HPV 55), while the others had HPV types other than the 37 types tested. The presence of HPV DNA was detected in two participants with white lesions and one participant with mixed red and white lesions. No high-risk HPV types were found in our samples.

## Discussion

We identified two types of betel nut chewing patterns in Guam and Saipan, which was consistent with previous findings [7]. In this and previous findings, similar variables were loaded and resulted in the 2-class model as the best fit. The variation in exposure to betel nut chewing (e.g., areca nut variety, nut maturity, components added) is considerable across populations. The exposure may be as simple as chewing the betel nut by itself [7], or as complex as chewing the nut with multiple ingredients [2]. The variation in betel nut chewing may influence exposure-disease associations, and thus it is important to capture in epidemiologic studies. The classification of chewers in this study was done to simplify modeling the complexity of betel nut chewing exposure, which may be modeled in other betel nut-disease associated studies where betel nut chewing exposure is variable.

The detection rate of OPMD was 15.3% in the first oral examination (by the hygienist in the field survey) and estimated as 12.4% after verification by the second oral examination (by the dentist in the dental office). Other studies have reported a prevalence of oral lesions and conditions of 12.7% in adults in southern Taiwan [25] and 22% (leukoplakia and oral submucous fibrosis) of high school students in the CNMI [13]. The prevalence of OPMD recorded in this study is consistent with the published prevalence data of OPMD (leukoplakia and OSF ) from several countries in the region [26]. US data from NHANES 111 Survey report [27] a much lower prevalence figure of oral leukoplakia, 0.66 ± 0.14% for males and 0.21 ± 0.05% in females and 0.42 ± 0.08% in both genders. The higher prevalence of OPMD in Micronesia is due to a range of OPMD including both leukoplakia and oral submucous fibrosis commonly found in this population attributable to their areca nut use. Contrasting life-style habits in Micronesia compared with the US population leads to a lower disease prevalence in the US.

Only one case, 0.3% of all chewers and 4.5% of those screened by the dentist, was diagnosed with oral cancer. This is close to 4.8% (514 oral cancers/10,657 screened) reported in a recent large prospective cohort study in Taiwan [28]. The oral cancer case and the majority of the other OPMD were among Class 2 chewers, despite Class 1 chewers chewing significantly longer (both in terms of years of betel nut use and duration of mastication). The evidence suggests that other ingredients included in Class 2 chewing behaviours, especially the addition of tobacco, may contribute (by synergy) to a higher disease incidence. The addition of slaked lime was a statistically significant chewing behaviour among Class 2 chewers; however, the IARC has previously concluded there was lack of evidence for carcinogenicity in experimental animals for slaked lime [2]. More epidemiologic research is needed to examine the potential causal effects of types of chewing behaviour on oral cancer incidence.

The proportion of Class 1 chewers who reported regular dental visits was significantly higher than Class 2 chewers (44.9% versus 24.3%), though both were lower than the overall Guam population of 54% [29]. Regular oral screening would be useful, especially among Class 2 chewers. Oral screening has been found to be cost-effective in high-risk populations in India [30] and by the U.S. estimates [31]. Compliance with referral to the secondary care was an issue for our study at 58.7 % and has been reported elsewhere with 63% compliance in Tower Hamlets, East London [32], 73% compliance in Kerala, India [33], and 52% in a similar study to ours using primary health care workers for screening in Sri Lanka [34]. We did not contact our patients who did not show up to find out reasons for non-compliance. Previous studies have reported ill health, economic reasons and work disdisturbance as main reasons for non-compliance following a screen detection [35]. Work is needed to encourage oral screening and compliance in Guam and Saipan. The innovative use of a mobile dental unit offered by Nunn and colleagues [32] as an outpost for screening may be worth exploring to reduce noncompliance in this community.

Seven of the 123 participants (5.7%) tested positively for HPV, although none were of the high- risk types. The low prevalence may be explained by our use of smears; HPV resides in the deeper layers of the epithelium and less could be harvested from exfoliating superficial layers. To the best of our knowledge, this is the first study to estimate oral HPV prevalence in betel nut chewers in this region. A systematic review has confirmed presence of HPV in oral biopsies and smears of OPMD [36]. The overall prevalence of high-risk HPV estimated from sentinel surveillance for cervical infection in the U.S. was 23% in 2003–2005 [37], and that of oral HPV in 2009–2010 was 6.9% [38]. HPV infection is the most prevalent sexually transmitted infection in the U.S., and 20% of the people with HPV are infected with more than one type [39]. The incidence of cervical cancer, a cancer attributed to HPV, was 9.5 per 100,000 for 2003–2007 in Guam [40]. The incidence of cervical cancer in the CNMI, which includes Saipan, was 69.1 per 100,000 for Chamorro females [41]. Therefore, genital HPV infection is common in the region, making the low rate of oral infection unexpected. The CNMI has taken a proactive approach to preventing HPV by offering HPV vaccination to young females students in the Public School System [42]. The bivalent HPV vaccinations protect against some of the high-risk HPV strains, including HPV 16 and 18 [43, 44], which were also found to cause cancer in the oral cavity and oropharynx [45].

As a pilot, this study is limited in design, thereby minimizing any inferences of association between betel nut chewing pattern and oral precancer risk. Another limitation is the noncompliance of participants with referral to the secondary oral examination, which may have affected the prevalence estimation of OPMD and oral cancer. However, the majority of the participants (58.7%) were compliant and there was good agreement between the first and second screenings. Despite the limitations, this study provides one of the first estimates of OPMD, oral cancer, and HPV infection in betel nut chewers in this region of the world - an oral health issue which carries a significant public health relevance, but has been understudied. Screening by dental care workers could save lives by detecting new cancers or precancer [46]. The classification of chewers in this study offers an innovative approach to simplifying the modeling of the complexity of betel nut chewing exposure. This technique may be useful in other betel nut-disease association studies with considerable exposure variability. The identification of high-risk behaviour among Class 2 chewers offers a way of targeting screening approaches (to that group) who are more likely to have OPMD.

## Conclusions

There were two types of betel nut chewing patterns identified in Guam and Saipan: Class 1 (predominantly areca nut users sometimes chewed with betel leaf – often ingested) and Class 2 (areca nut often chewed with betel leaf, lime, and tobacco – often discarded), with the majority (74%) being Class 2 chewers. The estimated prevalences were: 12.4% to 15.3% for OPMD, 0.3% for oral cancer, and 5.7% for HPV. The screening model used by us in this pilot study to recruit participants in Saipan was effective and appropriate but required modification for Guam as the prevalence of betel quid chewers was much lower and needed additional methods of recruitment. Additional epidemiologic studies are needed to examine the potential causal effects of specific types of chewing behaviour on oral cancer incidence. In the meantime, oral screening, especially among Class 2 chewers, and innovative strategies to reduce noncompliance to attend secondary facilities are encouraged.

## Abbreviations

CI: Confidence interval
CNMI: Commonwealth of the Northern Mariana Islands
DNA: Deoxyribonucleic acid
HPV: Human Papillomavirus
IARC: International Agency for Research on Cancer
SD: Standard deviation
WHO: World Health Organisation.

## References

[1] Gupta PC, Warnakulasuriya S (2002). Global epidemiology of betel nut usage. Addict Biol.

[2] International Agency for Research on Cancer (2004). Betel-Quid and Areca-nut Chewing and Some Areca-nut-Derived Nitrosamines.

[3] International Agency for Research on Cancer (1998). Tobacco Habits other than Smoking; Betel - Quid and Areca-Nut Chewing; and some Related Nitrosamines.

[4] Warnakulasuriya S, Johnson NW, van der Waal I (2007). Nomenclature and classification of potentially malignant disorders of the oral mucosa. J Oral Pathol Med.

[5] Warnakulasuriya S (2007). Oral precancer: terminology, diagnosis, and prognosis. The Inside Summit on Oral Cancer Discovery and Management.

[6] Paulino YC (2010). Describing and measuring variability of Areca catechu (betel nut) chewing in Micronesian populations in Guam.

[7] Paulino YC, Novotny R, Miller MJ, Murphy SP (2011). Areca (betel) nut chewing practices in Micronesian Populations. Hawaii Journal of Public Health.

[8] Katz AR, Palafox NA, Johnson DB, Yamada S, Ou AC, Minami JS (2004). Cancer epidemiology in the freely associated U.S. Pacific Islands jurisdictions: challenges and methodologic issues. Pac Health Dialog.

[9] Haddock RL (2005). Oral cancer incidence disparity among ethnic groups on Guam. Pac Health Dialog.

[10] Haddock R, Hoffman JH, Williams WR (1981). Betel nut chewing on Guam. Fiji Medical Journal.

[11] Pacific Islands Nutrition, No.49 (2001). Betel Chewing and Cancer.

[12] Government of the Republic of Palau (2011). RPPL no. 8–27 An Act to Adopt Measures Consistent With the Objective of the World Health Organization’s Framework Convention on Tobacco Control (FCTC), to Which the Republic is a Party, to Protect Present and Future Generations Against the Devastating Health, Social, Environmental and Economic Consequences of Tobacco Consumption and Exposure to Tobacco Smoke; to Amend, Repeal and Replace Sections of Chapter l 0 of Title ll of the Palau National Code and for Other Related Purposes.

[13] Oakley E, Demaine L, Warnakulasuriya S (2005). Areca (betel) nut chewing habit among high-school children in the Commonwealth of the Northern Mariana Islands (Micronesia). In Bull World Health Organ.

[14] Novotny R, Coleman P, Tenorio L, Davison N, Camacho T, Ramirez V, Vijayadeva V, Untalan P, Tudela MD (2007). Breastfeeding is associated with lower body mass index among children of the Commonwealth of the Northern Mariana Islands. J Am Diet Assoc.

[15] Paulino YC, Patricia C, Davison NH, Lee SK, Camacho TB, Tenorio LF, Murphy SP, Novotny R (2008). Nutritional characteristics and body mass index of children in the Commonwealth of the Northern Mariana Islands. J Am Diet Assoc.

[16] **World Health Organization review of areca (betel) Nut and tobacco use in the pacific: a technical report** http://www.wpro.who.int/tobacco/documents/201203_Betelnut/en/

[17] Paulino YC, Novotny R, Katz AR, Wilkens LR, Hurwitz EL (2013). Development and Validation of an Areca (Betel) nut Usage Measurement Tool for Micronesia [Abstract]. Proceedings of the 6th American Association for Cancer Research Conference on the Science of Cancer Health Disparities in Racial/Ethnic Minorities and the Medically Underserved; 2013 Dec 6–9; Atlanta, GA.

[18] Landis JR, Koch GG (1977). The measurement of observer agreement for categorical data. Biometrics.

[19] Warnakulasuriya S (2010). Screening for Oral Cancer and Precancer. University of Guam Presentation.

[20] Hernandez BY, McDuffie K, Zhu X, Wilkens LR, Killeen J, Kessel B, Wakabayashi MT, Bertram CC, Easa D, Ning L, Boyd J, Sunoo C, Kamemoto L, Goodman MT (2005). Anal human papillomavirus infection in women and its relationship with cervical infection. In Cancer Epidemiol Biomarkers Prev.

[21] Gravitt P, Peyton CL, Alessi TQ, Wheeler CM, Coutlee F, Hildesheim A, Schiffman MH, Scott DR, Apple RJ (2000). Improved amplification of genital human papillomaviruses. J Clin Microbiol.

[22] Gravitt PE, Peyton CL, Apple RJ, Wheeler CM (1998). Genotyping of 27 human papillomavirus types by using L1 consensus PCR products by single hybridization, reverse line blot detection method. J Clin Microbiol.

[23] McCutcheon AL (1987). Latent Class Analysis.

[24] Muthen B, Marcoulides GA, Schumacker RE (2001). Latent Variable Mixture Modeling. New Developments and Techniques in Structural Equation Modeling.

[25] Chung CH, Yang YH, Wang TY, Shieh TY, Warnakulasuriya S (2005). Oral precancerous disorders associated with areca quid chewing, smoking, and alcohol drinking in Southern Taiwan. J Oral Pathol Med.

[26] Lee C-H, Ko AM, Warnakulasuriya S, Yin BL, Sunarjo, Zain RB, Ibrahim SO, Liu ZW, Li WH, Zhang SS, Kuntoro, Utomo B, Rajapakse PS, Warusavithana SA, Razak IA, Abdullah N, Shrestha P, Kwan AL, Shieh TY, KO YC, Chen MK (2011). Intercountry prevalences and practices of betel-quid use in south, southeast and eastern Asia regions and associated oral preneoplastic disorders: an international collaborative study by Asian betel-quid consortium of south andeast Asia. Int J Cancer.

[27] Scheifele C, Reichart PA, Dietrich T (2003). Low prevalence of oral leukoplakia in a representative sample of the US. population. Oral Oncol.

[28] Lin W-J, Jiang R-S, Wu S-H, Chen F-J, Liu S-A (2011). Smoking, alcohol, and betel quid and OralCancer: a prospective cohort study. In J Oncol.

[29] Centers for Disease Control and Prevention (CDC): **Behavioral risk factor surveillance system survey data.** http://apps.nccd.cdc.gov/brfss/display.asp?cat=OH&yr=2012&qkey=8471&state=GU

[30] Subramanian S, Sankaranarayanan R, Bapat B, Somanathan T, Thomas G, Mathew B, Vinoda J, Ramadas K (2009). Cost-effectiveness of oral cancer screening: results from a cluster randomized controlled trial in India. Bull World Health Organ.

[31] Dedhia RC, Smith KJ, Johnson JT, Roberts M (2011). The cost-effectiveness of community-based screening for oral cancer in high-risk males in the united states: a Markov decision analysis approach. Laryngoscope.

[32] Nunn H, Lalli A, Fortune F, Croucher R (2009). Oral cancer screening in the bangladeshi community of tower hamlets: a social model. Br J Cancer.

[33] Sankaranarayanan R, Ramadas K, Thomas G, Muwonge R, Thara S, Mathew B, Rajan B, Trivandrum Oral Cancer Screening Study Group (2005). Effect of screening on oral cancer mortality in Kerala, India: a cluster-randomised controlled trial. Lancet.

[34] Warnakulasuriya KA, Ekanayake AN, Sivayoham S, Sivayoham S, Stjernswärd J, Pindborg JJ, Sobin LH, Perera KS (1984). Utilization of primary health care workers for early detection of oral cancer and precancer cases in Sri Lanka. Bull World Health Organ.

[35] Warnakulasuriya S, Ekanayake A, Stjernsward J, Pindborg JJ, Sivayoham S (1988). Compliance following referral in the early detection of oral cancer and precancer in Sri Lanka. Community Dent Oral Epidemiol.

[36] Syrjänen S, Lodi G, von Bültzingslöwen I, Aliko A, Arduino P, Campisi G, Challacombe S, Ficarra G, Flaitz C, Zhou MH, Maeda H, Miller C, Jontell M (2011). Human papillomaviruses in oral carcinoma and oral potentially malignant disorders: a systematic review. Oral Dis.

[37] Centers for Disease Control and Prevention (CDC): **Incidence, prevalence, and cost of sexually transmitted infections in the United States.** http://www.cdc.gov/std/stats/STI-Estimates-Fact-Sheet-Feb-2013.pdf

[38] Gillison ML, Broutian T, Pickard RK, Tong ZY, Xiao W, Kahle L, Graubard BI, Chaturvedi AK (2012). Prevalence of oral HPV in the United States, 2009–2010. J Amer Medl Assoc.

[39] Centers for Disease Control and Prevention (2012). Sexually Transmitted Disease Surveillance 2011.

[40] Guam Comprehensive Cancer Control Coalition (2011). Guam Cancer Facts and Figures 2003–2007 (Revised February 2011).

[41] Asian and Pacific Islander American Health Forum: **Chamorros in the United States.** http://www.hawaii.edu/hivandaids/Chamorros_In_The_United_States.pdf

[42] Sablan M (2008). Report on the HPV Campaign in the high schools of the Northern Mariana Islands (CNMI) [Abstract].

[43] U.S. Food and Drug Administration: **Vaccines, blood & biologics.** http://www.fda.gov/BiologicsBloodVaccines/Vaccines/ApprovedProducts/ucm186957.htm

[44] U.S. Food and Drug Administration: **Vaccines, blood & biologics.** http://www.fda.gov/BiologicsBloodVaccines/Vaccines/ApprovedProducts/ucm094042.htm

[45] International Agency for Research on Cancer (2007). Human Papillomaviruses.

[46] Kerr AR (2000). Life saving oral cancer screening. N Y State Dent J.

[CR47] The pre-publication history for this paper can be accessed here: http://www.biomedcentral.com/1472-6831/14/151/prepub

